# Neuregulin 1 Reduces Motoneuron Cell Death and Promotes Neurite Growth in an *in Vitro* Model of Motoneuron Degeneration

**DOI:** 10.3389/fncel.2017.00431

**Published:** 2018-01-09

**Authors:** Guillem Mòdol-Caballero, Daniel Santos, Xavier Navarro, Mireia Herrando-Grabulosa

**Affiliations:** ^1^Department of Cell Biology, Physiology and Immunology, Institute of Neurosciences, Universitat Autònoma de Barcelona, Bellaterra, Spain; ^2^Centro de Investigación Biomédica en Red sobre Enfermedades Neurodegenerativas (CIBERNED), Bellaterra, Spain

**Keywords:** neuregulin 1, ErbB receptors, motoneuron, excitotoxicity, spinal cord, organotypic culture, amyotrophic lateral sclerosis, neurite growth

## Abstract

Amyotrophic Lateral Sclerosis (ALS) is a devastating neurodegenerative disorder with no effective treatment currently available. Although the mechanisms of motoneuron (MN) death are still unclear, glutamate excitotoxicity and neuroinflammatory reaction are two main features in the neurodegenerative process of ALS. Neuregulin 1 (NRG1) is a trophic factor highly expressed in MNs and neuromuscular junctions. Several recent evidences suggest that NRG1 and their ErbB receptors are involved in ALS. However, further knowledge is still needed to clarify the role of the NRG1-ErbB pathway on MN survival. In this study we used an *in vitro* model of spinal cord organotypic cultures (SCOCs) subject to chronic excitotoxicity caused by DL-*threo*-β-hydroxyaspartic acid (THA) to characterize the effect of NRG1 on MN survival. Our results show that addition of recombinant human NRG1 (rhNRG1) to the medium significantly increased MN survival through the activation of ErbB receptors which was ablated with lapatinib (LP), an ErbB inhibitor, and reduced microglial reactivity overcoming the excitotoxicity effects. rhNRG1 activated the pro-survival PI3K/AKT pathway and restored the autophagic flux in the spinal cord culture. Moreover, addition of rhNRG1 to the medium promoted motor and sensory neurite outgrowth. These findings indicate that increasing NRG1 at the spinal cord is an interesting approach for promoting MN protection and regeneration.

## Introduction

Amyotrophic lateral sclerosis (ALS) is the most common form of motoneuron (MN) disease, characterized by the loss of MNs of primary motor cortex, brainstem and spinal cord (Wijesekera and Leigh, 2009). Most patients with ALS die within 3–5 years after symptoms onset due to respiratory failure (Robberecht and Philips, 2013). Unfortunately, there is no therapy for this disease, and the only drugs approved for use in ALS, riluzole and edaravone, only slightly prolong patients’ survival. The etiopathogenesis of ALS remains to be elucidated, but it has been proposed that a complex interplay between excitotoxicity, neuroinflammation, oxidative stress, protein aggregation and mitochondrial dysfunction contribute to MN degeneration (Robberecht and Philips, 2013; Mancuso and Navarro, 2015). Among these pathogenetic mechanisms, excitotoxicity is considered as a firm mechanism involved in the disease because of data obtained from ALS patients and animal and cellular models as well as inferred by the therapeutic effect of riluzole, an antiglutamatergic drug. The importance of excitotoxicity in MN death has been demonstrated in ALS mouse models in which excessive extracellular glutamate levels contribute to MN loss (Van Damme et al., 2005). Several *in vitro* models have been used to study glutamate neurotoxicity. Among them, the spinal cord organotypic culture (SCOC) offers advantages for assessing MN degeneration and potential therapeutic agents (Herrando-Grabulosa et al., 2016). This model is based on the selective inhibition of glutamate transport which continuously raises the concentration of glutamate in the culture medium, resulting in a slow degeneration of spinal MNs over several weeks (Rothstein et al., 1993).

Neurotrophic factors derived from alternatively spliced forms of the neuregulin 1 (NRG1) gene have been shown to play an important role in peripheral nerve development and regeneration, myelination, maintenance of neuromuscular junctions, and also on microglial activation in peripheral nerve diseases, whereas ablation of NRG1 impairs axonal regeneration (Mei and Nave, 2014). NRG1 acts through the EGF domain of ErbB receptors, a family of tyrosine kinase transmembrane receptors. NRG1 is localized in the MN endomembrane system, destined to be anterogradely transported either for axon-Schwann cell signaling or for delivery to the neuromuscular junction. Interestingly, NRG1 has been found present within the subsurface cistern in postsynaptic sites of cholinergic C terminals apposed to spinal MNs (Issa et al., 2010; Gallart-Palau et al., 2014). ErbB2 and ErbB4 receptors are present in the presynaptic compartment, suggesting that NRG1 acts as a retrograde signaling molecule in MNs synaptic connections. A role of NRG1 expressed in C synaptic boutons was recently suggested for ALS, since spinal MNs showed a transient increase of NRG1 during disease progression in SOD1^G93A^ mice, whereas oculomotor MNs, which are spared in ALS, lack both C boutons and associated NRG1 (Gallart-Palau et al., 2014). In fact, it was recently reported that viral-mediated delivery of type III-NRG1 to the spinal cord restored the number of C-boutons and slightly extend the survival of SOD1 transgenic mice (Lasiene et al., 2016).

Alterations in the NRG1/ErbB system have been related to MN degeneration and ALS. Loss-of-function mutations on the NRG1 receptor ErbB4 produce late-onset, autosomal-dominant ALS in human patients (Takahashi et al., 2013). NRG1 type III expression was found reduced in both ALS patients and SOD1^G93A^ mice in parallel with MN loss, but NRG1 type I was increased and associated with glial activation (Song et al., 2012). We recently reported that increased expression of NRG1 in skeletal muscle promotes collateral reinnervation and neuromuscular junction maintenance in the SOD1^G93A^ mouse model of ALS (Mancuso et al., 2016).

Our goal in this study was to evaluate the role of NRG1 in the SCOC subjected to chronic excitotoxicity, in order to assess the potential effects of exogenous NRG1 on MN survival and regeneration, and its mechanisms of action.

## Materials and Methods

### Spinal Cord Organotypic Cultures for Assessment of Neuroprotection

SCOCs were prepared on the basis of the method previously described (Rothstein et al., 1993). The experimental procedure was approved by the Ethics Committee of Universitat Autònoma de Barcelona and followed the European Communities Council Directive 2010/63/EU. P8 Sprague-Dawley rats were used in this study. After euthanasia, the spinal cord was aseptically harvested and placed in ice-cold high glucose-containing (6.4 mg/mL) Gey’s Balanced Salt Solution (GBSS; Sigma-Aldrich, St. Louis, MO, USA), and meninges were removed. The spinal cord was cut transversely in 350 μm thick slices using a McIlwainTissue Chopper (The Mickle Laboratory Engineering Co., Surrey, UK).

To investigate MN survival, glial reactivity and the ErbB and signaling pathways activation SCOCs were obtained from at least three independent cultures performed at different days and resulting in 12 slices for condition. L4-L5 lumbar sections were carefully transferred on Millicell-CM porous membranes (0.4 μm; Millipore,Burlington, MA, USA) into a six-well plate containing 1 mL of incubation medium (50% minimal essential medium (MEM), 25 mM Hepes, 25% heat-inactivated horse serum, 2 mM glutamine, and 25% Hank’s Balanced Salt Solution (HBSS) supplemented with 25.6 mg/ml glucose; pH 7.2). Cultures were let to stabilize for 1 week. During the first week in a SCOC, a high number of neurons die naturally and glial cells show strong reactivity due to the axotomy performed during the culture procedure, which later stabilize. Then DL-*threo*-β-hydroxyaspartic acid (THA; 100 μM) was added to induce chronic excitotoxicity (Corse et al., 1999). Concomitantly, some slices were treated with recombinant human NRG1 (rhNRG1; 100 ng/ml) or with lapatinib (LP), an ErbB blocker, at two different concentrations (6 μM and 12 μM). PRE084 (10 μM) a sigma-1 receptor agonist that promotes neuroprotection and neurite elongation was used as a positive control (Guzmán-Lenis et al., 2009). THA and the treating compounds were renewed at each medium exchange, twice per week until 14 days *in vitro* (DIV) for western blot analysis and histological stainings, or to 28 DIV for MN survival and microglial reactivity analyses. In this model of chronic excitotoxicity, THA induces around 40% of MN death after 3 weeks of treatment (28 DIV; Rothstein et al., 1993), whereas microglial activation occurs after 2 weeks of THA treatment (21 DIV) and is maintained at 28 DIV (Lee et al., 2012).

### Protein Extraction and Western Blot

SCOC slices were prepared for protein extraction and homogenized in modified RIPA buffer (50 mM Tris–HCl pH 7.5, 1% Triton X-100, 0.5% sodium dodecyl sulfate (SDS), 100 mM NaCl, 1 mM EDTA) adding 10 μl/ml of Protease Inhibitor cocktail (Sigma) and PhosphoSTOP phosphatase inhibitor cocktail (Roche). Twenty to forty microgram of protein of each sample were loaded in SDS-polyacrylamide gels at different percentages (7.5%–15%). For heavy proteins (ErbB receptors) the western blot transfer was applied 3.5 h at room temperature with constant intensity of 360 mA. For the rest of the proteins the transfer was made 1 h at room temperature with constant voltage of 90 V. The membranes were blocked with 5% BSA in TBS plus 0.1% Tween-20 for 1 h, and then incubated with primary antibodies at 4°C overnight. The primary antibodies used were: anti-NRG1 (1:200, sc-228916, Santa Cruz Biotechnology), anti-ErbB4 (1:500, 4795S, Cell Signaling), anti-ErbB3 (1:500, 12708S, Cell Signaling), anti-ErbB2 (1:500, 4290S, Cell Signaling), anti-pErbB4 (1:500, 4757S, Cell Signaling), anti-pErbB3 (1:500, 2842S, Cell Signaling), anti-pErbB2 (1:500, 2243L, Cell Signaling), anti-Akt (1:1000, 4691S, Cell Signaling), anti-pAkt (1:1000, 4060, Cell Signaling), anti-ERK1/2 (1:500, 4348, Cell Signaling), anti-pERK1/2 (1:500, 9106, Cell Signaling), anti-LC3 (1:500, ab51520, Abcam), anti-p62 (1:500, 610833, BD Biosciences), anti-Beclin 1 (1:1000, ab62557, Abcam), anti-Actin (1:10,000, A5316, Sigma) and anti-GAPDH (1:10,000, MAB374, Millipore). Horseradish peroxidase-coupled secondary antibody (1:3000; Vector Laboratories, Burlingame, CA, USA) incubation was performed for 1 h at room temperature. The membranes were visualized using enhanced chemiluminescence method and the images were collected using a Chemidoc apparatus. Western blots were then analyzed using the Lane and Band plugin from the Image Lab software (BioRad), and normalized first by the loading control (actin and GAPDH) and afterwards by each control sample. Each sample analyzed was extracted from four slices and 3–7 samples were analyzed per each treatment condition.

### Immunoflurorescence Analyses

We fixed slices in the different experimental conditions with 4% paraformaldehyde in PBS for 1 h at RT. After blocking with 5% normal horse serum (Vector Laboratories, Burlingame, CA, USA) and 0.3% Triton-X-100 in TBS (TBS-TX), we incubated the sections for 48 h with primary antibodies against anti-neurofilament H non-phosphorylated (SMI-32, 1:1000, BioLegend) or anti-ionized calcium binding adapter molecule 1 (Iba-1, 1:1000; Wako, Japan). Slices were thoroughly washed in TBS with 0.1% Tween-20 (TBS-T) and incubated with the appropriate secondary antibody Alexa Fluor^®488^ donkey anti-rabbit IgG (1:500) and Alexa Fluor^®594^ donkey anti-mouse IgG (1:500; Invitrogen, Carlsbad, CA, USA), diluted in TBS-T for 2 h. Finally, cell nuclei were labeled with DAPI (1:2000) for 1 min in TBS and the sections mounted with Fluoromount-G medium (SouthernBiotech, Birmingham, AL,USA). We analyzed the slides under confocal microscopy, and counted the SMI-32 positive cells in each hemislice using the tool Cell Counter from ImageJ software (NIH)^1^. MNs were selected according to the following criteria: localization in ventral horns and polygonal shape, with clear dendrites. To quantify the microglia, we selected the ventral zone using for each image the same area, and then quantified the integrated density (area of the ROI × mean of the ROI) using the ROI manager tool of ImageJ.

### Immunohistochemical Analyses

Slices were fixed with 4% paraformaldeyde in PBS for 24 h at 4°C, and then, cryoprotected in 30% sucrose in PBS and stored at 4°C. Cyroprotected slices were then sectioned with a cryostat (Leica) into 6–8 10 μm thick sections from each SCOC. The endogenous peroxidase activity was inhibited (70% Methanol, 30% TBS 1X, 2% H_2_O_2_) and thereafter a blocking solution (5% normal horse serum and 1% BSA in TBS-T) was added. We incubated the slides overnight at 4°C with primary antibodies against anti-ErbB4 (1:100, 4795S, Cell Signaling) and anti-ErbB2 (1:100, 4290S, Cell Signaling). Slides were washed with TBS-T and incubated with a secondary antibody horse anti rabbit HRP conjugate (Vector Laboratories, USA) overnight at 4°C. Afterwards, we incubated the slides with the VECTASTAIN^®^ Elite^®^ ABC complex (Vector Laboratories, USA) for 1 h at RT and a DAB solution (Vector Laboratories, USA) was used for brown color development. We finally counterstained the slides with cresyl violet to localize the MNs in the ventral horn.

### Organotypic Cultures for Assessment of Neurite Growth

For assessing neurite outgrowth, we used spinal cord sacral sections and also dorsal root ganglia (DRG) explant cultures (*n* = 11/group) embedded in a collagen gel, as previously described (Allodi et al., 2011; Santos et al., 2016). Collagen type I solution (#354236, Corning) at a concentration of 3.83 mg/ml was mixed with basal Eagle’s medium (Gibco) and 7.5% of sodium bicarbonate solution. NRG1 at a concentration of 100 ng/ml was added to the collagen gel in the treated slices, whereas the same volume of PBS was used for the control slices. Single 30 μl drops were deposited on poly-D-lysine (1 g/ml, Sigma) coated coverslips, which were placed in 24-well multidishes (Iwaki, Asahi Technoglass, Chiba, Japan) and kept in the incubator for 2 h to induce collagen gel formation. Spinal cord slices and DRG explants were then embedded in the gelled collagen droplets, and placed in the incubator for 45 min before adding Neurobasal medium (NB, Invitrogen), supplemented with B27 (Invitrogen), glutamine and penicillin/streptomycin (Sigma). After 1 day in culture, the medium of spinal cord cultures was removed and changed by a penicillin/streptomycin free medium. DRG explants were cultured for 2 days, and spinal cord slices for 4 days.

### Neurite Growth Analysis

Spinal cord and DRG cultures were fixed with 4% paraformaldehyde in PBS for 30 min. Afterwards, the samples were incubated for 48 h with primary antibody mouse RT97 that recognizes phosphorylated epitopes of neurofilaments (1:200, Developmental Studies Hybridoma Bank) at 4°C, washed and incubated with secondary antibody AF594 conjugated donkey anti-mouse (1:200, Jackson IR) overnight at 4°C. After two washes samples were mounted on slides with Mowiol containing DAPI (1:10,000, Sigma) nuclear counterstain.

Cultures were visualized with an Olympus BX51 fluorescence microscope, images of different areas were taken with Cell A software (Olympus) and merged using Adobe Photoshop CS3 (Adobe System). Whole culture images were analyzed with the Neurite-J plug-in (Torres-Espín et al., 2014) for ImageJ software and the number of neurites grown at 50 μm intervals from the explant was compared between sets of cultures. The length measured for each neurite was the distance from the end of the neurite straight back to the DRG body radially (Deister and Schmidt, 2006) or to the neuronal body in spinal cord slices (*n* = 10–15 per slice).

### Data Analysis

Data were evaluated using GraphPad Prism 5 software (San Diego, CA, USA). For statistical analysis, immunofluorescence data were analyzed by one-way ANOVA followed by Bonferroni multiple *post hoc* comparisons test. Western blot quantification results were analyzed by two-tail unpaired *t*-test for two groups comparison and one-way ANOVA followed by Tukey multiple *post hoc* test for multiple group comparison. Data were expressed as mean ± SEM. For the neurite length assessment, results were analyzed by performing a two-tailed unpaired *t*-test. All differences were considered statistically significant when *p* < 0.05.

## Results

### rhNRG1 Prevents Motoneuron Cell Death

**Figure 1:**
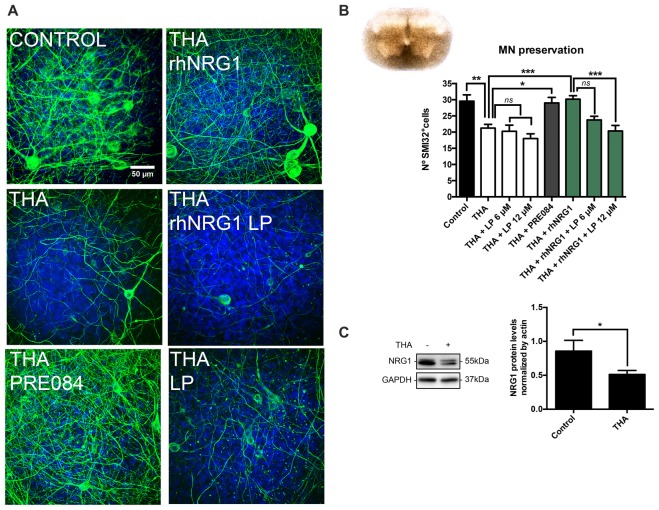
Recombinant human NRG1 (rhNRG1) promotes motoneuron (MN) survival in the spinal cord organotypic culture (SCOC) under excitotoxicity. **(A)** Representative microphotographs of MNs in the ventral horn of the slices labeled with the SMI-32 antibody and DAPI at 28 days in vitro (DIV). Cultures were subjected to excitotoxicity by DL-threo-b-hydroxyaspartic acid (THA) alone or co-treated with rhNRG1 with or without lapatinib (LP) inhibitor, or with PRE084, a sigma 1 receptor agonist used as a positive control. Scale bar = 50 μm. **(B)** Microphotograph of a spinal cord slice in culture at 28 DIV is shown. Histogram graph showing the number (mean ± SEM, *n* = 24–31 hemisections per treatment) of SMI-32 positive cells in the ventral horn of each spinal cord slice. ***p < 0.001; **p < 0.01; *p < 0.05. **(C)** Western blot of the Neuregulin 1 (NRG1) protein in SCOCs under control or THA condition at 14 DIV. Bar graph showing the mean ± SEM protein levels of NRG1 (*n* = 3 cultures per condition). *p < 0.05.

In SCOCs prepared for the assessment of neuroprotection, addition of THA in the medium significantly reduced the number of SMI32 positive cells in the ventral horn (21 ± 1) compared to the control slices (30 ± 2; Figures 1A,B). We also evaluated by western blot the endogenous level of NRG1 in the SCOCs after chronic excitotoxicity, and found that after 21 days of exposure to THA, NRG1 levels were significantly reduced (0.51 ± 0.05) compared to control cultures (0.85 ± 0.15; Figure 1C). With the aim to investigate if NRG1 could act as a neuroprotective agent for spinal cord MNs, we added 100 ng/mL of rhNRG1 to the SCOCs exposed to THA, and found significant preservation of MNs (30 ± 1) at similar level than with addition of PRE084 (29 ± 2; Figures 1A,B). The neuroprotective effect induced by rhNRG1 alone was abolished by simultaneous treatment with LP, an ErbB blocker, at 12 μM (20 ± 2) and partially at 6 μM (24 ± 1; Figure 1B). Addition of LP alone at 6 μM and 12 μM had no influence on the loss of MNs induced by THA (20 ± 2 and 18 ± 2, respectively). These results indicate that rhNRG1 induces protection to MNs under an excitotoxic chronic insult through ErbB receptors.

### rhNRG1 Signaling under Chronic Excitotoxicity

**Figure 2:**
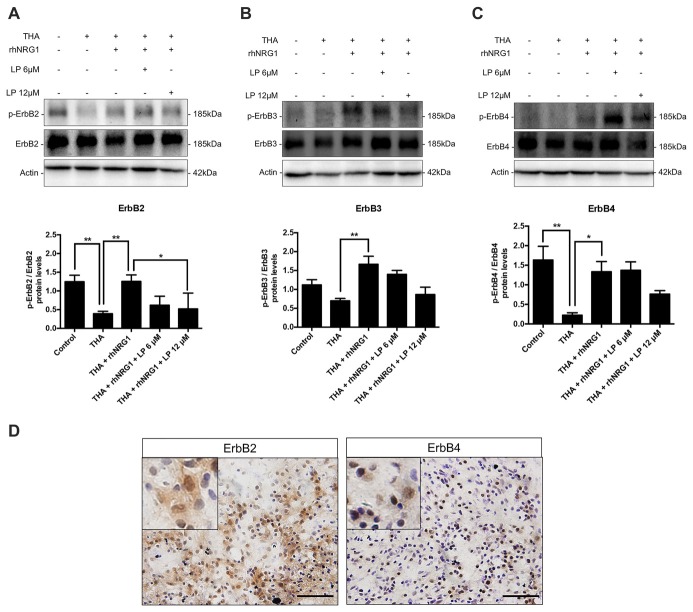
ErbB receptors activation. Western blot of SCOCs revealed an increased phosphorylation ratio of ErbB2 **(A)**, ErbB3 **(B)**, and ErbB4 **(C)** receptors upon rhNRG1 treatment, that was totally or partially blocked by co-addition of LP. Data are shown as mean ± SEM with *n* = 3–5 values per treatment. **p < 0.01; *p < 0.05. **(D)** Microphotographs of ErbB2 and ErbB4 protein labeling with DAB and cresyl violet in control SCOCs at 14 DIV. Inset show MNs labeled against ErbB receptor at higher magnification. Scale bar = 100 μm.

In order to evaluate the signaling pathways underlying the neuroprotective effect of rhNRG1, we performed immunoblot analyses of the ErbB receptors. The total isoform expression of ErbB2, 3 and 4 receptors was not significantly modified in the different SCOC conditions as measured by immunoblot. However, excitotoxicity caused a significant reduction of the proportion of activated ErbB2 and ErbB4 and not significant for ErbB3 in comparison with control cultures (Figure 2). The activation of ErbB receptors was determined by calculating the ratio between the phosphorylated vs. the total protein levels. Interestingly, the level of phosphorylated ErbB receptors was restored by addition of rhNRG1 to the culture medium (Figure 2). On the other hand, addition of LP at 12 μM significantly inhibited the activation of ErbB2 induced by rhNRG1, and tended to block although not significantly the levels of phosphorylated ErbB3 and ErbB4 receptors (Figure 2). In addition, histological staining was performed to localize the ErbB receptors in the ventral horn of control SCOC. We confirm that ErbB2 and ErbB4 are expressed in MNs in the spinal cord slices taken at 14 DIV. The ErbB2 expression pattern was similar to that in intact, non-cultured, spinal cord. ErbB4, which is located in the cytosol of the MNs, appeared partly distributed to the nucleus after the axotomy produced for the culture (Figure 2D).

**Figure 3:**
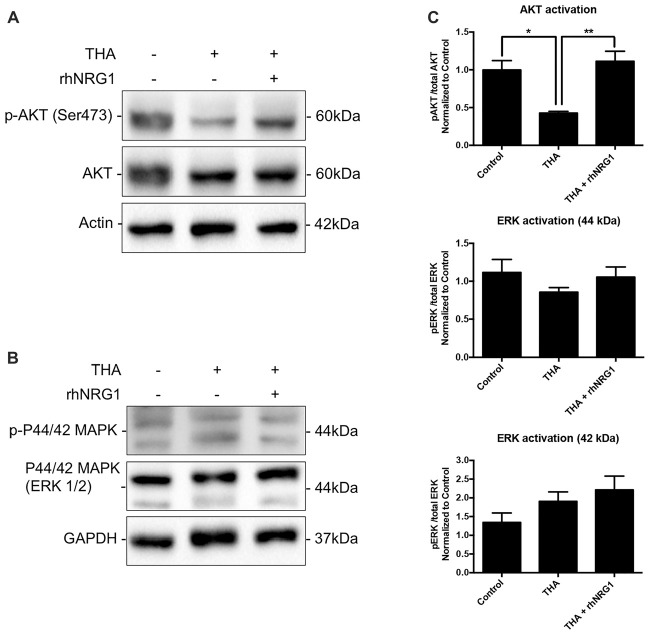
Activation of downstream targets by rhNRG1. **(A,C)** Western blot of SCOCs revealed increased phosphorylation of AKT upon rhNRG1 treatment compared to the excitotoxic treatment alone. **(B,C)** No significant differences for ERK activation. Data are shown as mean ± SEM with *n* = 3–5 values per treatment. **p < 0.01; *p < 0.05.

**Figure 4:**
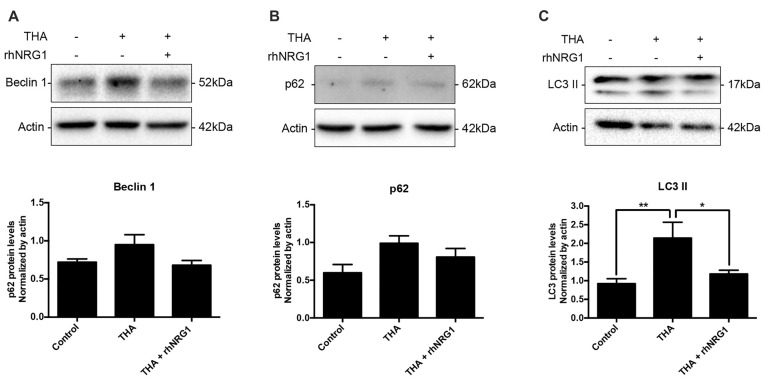
Autophagic flux modulation by rhNRG1. Western blot of SCOCs did not show significant changes for Beclin 1 **(A)** and p62 **(B)**. However, THA induced an increase of autophagosome marker LC3II **(C)**, which was prevented when rhNRG1 was applied to the SCOCs. Data are shown as mean ± SEM with *n* = 3–7 values per treatment. **p < 0.01; *p < 0.05.

To corroborate the functional activation of the ErbB receptors through NRG1 signaling, we analyzed changes in the phosphorylation levels of AKT and ERK1/2, as downstream targets of ErbB related to neuronal survival. Increased activation of AKT by rhNRG1 treatment was evidenced by the significantly higher ratio of the phosphorylated vs. the total form of the protein compared to addition of THA alone (Figures 3A,C). In contrast, we did not find significant changes in the activation of ERK1/2 (Figures 3B,C). Because AKT pathway can modulate macroautophagic mechanisms, we examined the expression of different markers. Upon rhNRG1 treatment, we found a reduction of Beclin 1, a phagophore formation marker, and of p62, a protein involved in the proteasomal degradation of ubiquitinated proteins, compared to the increase produced by THA treatment alone, although the differences were not significant (Figures 4A,B). Interestingly we found differences in the LC3-II, a marker of autophagosome formation. Under chronic excitotoxicity the LC3-II levels were enhanced, whereas addition of rhNRG1 maintained the levels of LC3-II similar to the control condition (Figure 4C).

### rhNRG1 Reduces Microglial Reactivity

**Figure 5:**
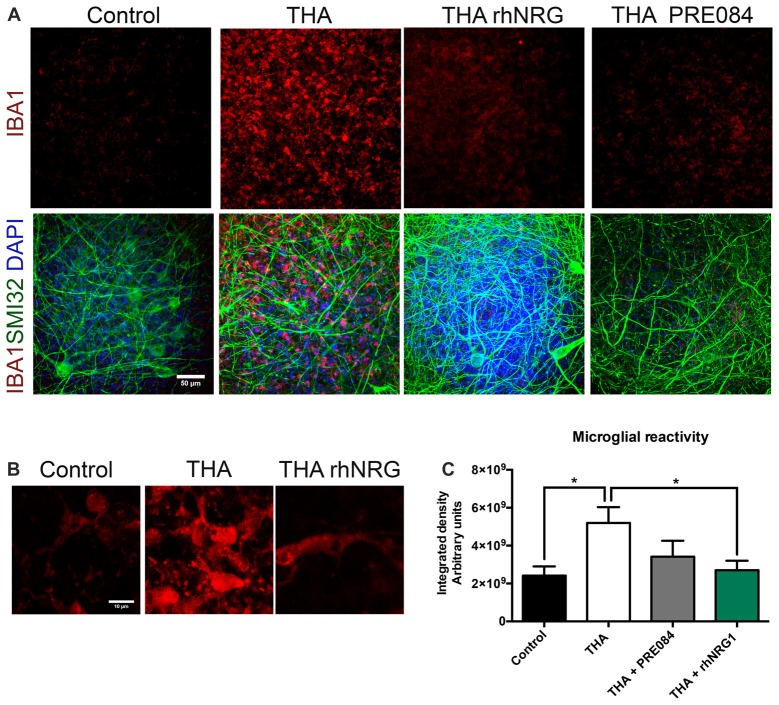
Reduction of microgliosis by rhNRG1. **(A)** Representative microphotographs showing microglia stained with anti-IBA1 (red), non-phosphorylated H neurofilament SMI32 (green) and DAPI (blue) at 21 DIV post-THA treatment alone or co-treated with rhNRG1 (100 ng/ml) or PRE084 (10 μM). Scale bar = 50 μm. **(B)** Higher magnification images of microglia stained with anti-IBA1 showing morphological differences between control, THA added and THA+ rhNRG1 conditions. Scale bar = 10 μm. **(C)** Bar graph showing the microglial reactivity measured by the integrated density in the ventral horn of SCOCs (mean ± SEM, *n* = 15 hemisections per treatment). *p < 0.05.

We also analyzed whether rhNRG1 had a role modulating the microglial activation in the SCOC. Microglial reactivity markedly increased with THA treatment, as revealed by the integrated density of Iba-1 labeling (5.19 × 10^9^ ± 8.30 × 10^8^) at 28 DIV (Figure 5). Addition of rhNRG1 under chronic excitotoxicity significantly reduced microgliosis (2.70 × 10^9^ ± 4.98 × 10^8^) at the same level than control slices (2.41 × 10^9^ ± 4.86 × 10^8^). PRE084 caused a less marked reduction of microglial reactivity that did not reach significance (3.41 × 10^9^ ± 8.40 × 10^8^).

### rhNRG1 Enhances Motor and Sensory Neurite Outgrowth

**Figure 6:**
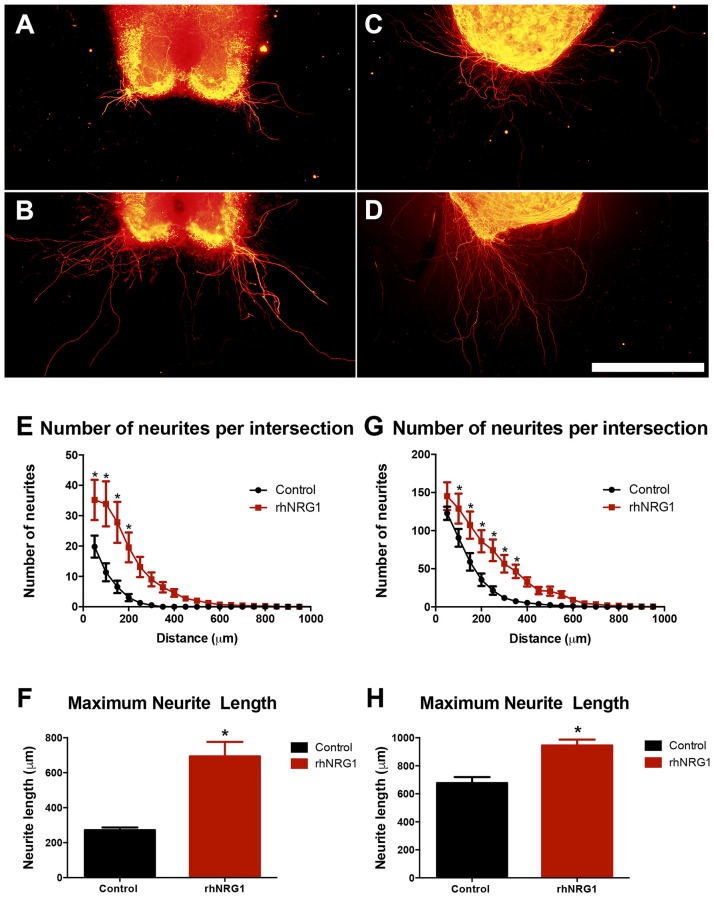
rhNRG1 enhances neurite outgrowth. Representative microphotographs of untreated and rhNRG1-treated SCOC **(A,B)** and dorsal root ganglia (DRG) **(C,D)** cultures embeeded in collagen. Graphs show the number of neurites per intersection and the maximum neurite length in the SCOC **(E,F)** and the DRG **(G,H)** cultures. Scale bar = 500 μm. Data are shown as mean ± SEM with *n* = 11 slices per treatment. *p < 0.05.

Because we observed in rhNRG1-treated slices a larger bundle of tangled axons than in control slices, we assessed whether rhNRG1 might influence neurite growth by using modified organotypic culture models (Figures 6A,B; Allodi et al., 2011). rhNRG1 treated SCOCs showed significantly higher number of growing motor neurites and increased average length of the longest neurites (694 ± 82 μm) compared to the untreated control cultures (271 ± 16 μm; Figures 6E,F). Additionally, we also evaluated the role of rhNRG1 on sensory neurite elongation (Figures 6C,D). rhNRG1-treated DRG also showed increased number of growing sensory neurites and length of the longest neurites (945 ± 42 μm) compared to control cultures (677 ± 42 μm; Figures 6G,H). However, the effect was less marked for sensory than for motor neurites.

## Discussion

The results of this study demonstrate that NRG1 exerts neuroprotective effects on MNs under chronic excitotoxicity, and also enhances neurite growth. These properties point to the NRG1-ErbB system as a potential target of interest for the treatment of MN degenerative diseases.

We used two relevant models of neural organotypic cultures in order to independently assess these two actions *in vitro*. The SCOC has the advantage to preserve the anatomical organization of the spinal cord, neuronal connectivity and glial–neuronal interactions. The SCOC model under chronic excitotoxicity induced by THA is an *in vitro* model based on the role of glutamate toxicity. It has been proved a useful *in vitro* model for screening potential therapeutic drugs (Guzmán-Lenis et al., 2009; Herrando-Grabulosa et al., 2016; Pandamooz et al., 2016) against MN degeneration that does not depend on known genetic alterations, thus being relevant for the majority of sporadic ALS cases and other MN diseases. The role of NRG1 and its isoforms on the ALS pathogenesis is still controversial. The studies investigating the expression of NRG1 and its ErbB receptors in the spinal cord have reported several alterations in samples of mouse models and ALS patients. Regarding the mRNA levels of NRG1, the Type III isoform was found decreased in the ventral horn of the spinal cord from ALS human samples as well as in SOD1^G93A^ transgenic mice in parallel with MN loss, whereas the Type I isoform was reported to increase at advanced stage of the disease in SOD1^G93A^ mice in one study (Song et al., 2012) and to decrease in another (Lasiene et al., 2016). In the SCOCs under chronic excitotoxicity we found low expression of NRG1 compared to control slices, and addition of exogenous NRG1 significantly preserved the number of surviving MNs in the ventral horn. The rhNRG1 corresponds to the EGF-domain of NRG1, which is able to activate ErbB receptors 2, 3 and 4. The EGF-domain of NRG1 can bind to ErbB3 and ErbB4 by inducing their homodimerization or the heterodimerization with ErbB2. As a consequence of the dimerization ErbB receptors phosphorylate. We showed that the neuroprotective effects of rhNRG1 are mediated by the activation of ErbB receptors because the addition of LP, an inhibitor of ErbB receptors, blocks the neuroprotective action of rhNRG1. In addition we showed that the ErbB2 and ErbB4 receptors are expressed in MNs, although we could not confirm the expression of ErbB3 due to limitations of the antibodies available. The ErbB2 pattern was the same as previously shown at *in vivo* samples (Song et al., 2012), while ErbB4 appeared in the nucleus of the MNs after the axotomy produced in the culture. Upon phosphorylation of ErbB receptors, the PI3K-AKT pro-survival pathway was activated as demonstrated by immunoblot. In cultures of embryonic rat MNs, NRG1 was shown to inhibit apoptosis during the period of embryonic programmed cell death by a PI3K-dependent pathway, although in this case it did not increase the relative level of p-AKT (Ricart et al., 2006). Despite the fact that ERK pathway is another cascade induced upon ErbB receptor activation, no differences of ERK1/2 were detected after rhNRG1 treatment.

Autophagy is another common feature in MN diseases that has already been described in the SCOC under chronic excitotoxicity (Matyja et al., 2005; Herrando-Grabulosa et al., 2013). However, the role of autophagy in promoting neuronal cell death or survival is a subject of debate. After THA treatment we found evidence of accumulation of autophagosomes that corresponds to an early stage of autophagy cell death induction. In contrast, addition of rhNRG1 to the culture decreased the levels of the phagopore formation marker Beclin 1 and the autophagosome marker LC3-II. Therefore, rhNRG1 may act restoring the autophagic flux by reducing the number of autophagosomes formed and starting p62 degradation in the lysosome after the fusion, avoiding accumulation in the cytosol.

MNs are preferentially damaged by the prolonged blockade of glutamate uptake produced by THA addition, causing excitotoxicity. In the SCOC this mechanism is mediated by non NMDA receptors, such as AMPA receptor (Rothstein et al., 1993). While these channels permit rapid Ca^2+^ entry, MNs buffer the consequent cytosolic Ca^2+^ load poorly (Lips and Keller, 1998), with the consequence that much of the Ca^2+^ is readily taken up into mitochondria, resulting in strong reactive oxidative species (ROS) generation (Carriedo et al., 2000; Rao et al., 2003). Furthermore, ROS may be able to exit the MN, disrupting glutamate transport in surrounding astrocytes, resulting in increased extracellular glutamate accumulation, and further propagation of the injury cascade (Rao et al., 2003; Yin and Weiss, 2012). Recently it has been shown that NRG-1β plays an important role in modulating Ca^2+^ homeostasis and preventing apoptosis through activating PI3K/AKT pathway in DRG sensory neurons subjected to excitotoxicity induced by glutamate (Liu et al., 2011). This evidence suggests that in the SCOC, rhNRG1 treatment may modulate Ca^2+^ homeostasis and thus reduce the excitotoxicity through this mechanism.

On the other hand, we used SCOCs and DRG explants embedded in a 3D collagen matrix, which creates a permissive environment for neurite elongation (Allodi et al., 2011; Santos et al., 2016), to assess the effects of NRG1 on neurite growth. Our results showed that rhNRG1 increased the number and length of the neurites emerging from both motor and sensory neurons in the SCOC and DRG cultures respectively. NRG1 plays an important role in myelination during development and also after injury in the peripheral nerve, and promotes the role of Schwann cells supporting axonal regeneration (Fricker and Bennett, 2011; Gambarotta et al., 2013; Stassart et al., 2013). Immediately after injury, the soluble NRG1 transcript is upregulated in the lesioned nerve, mostly released by reactive Schwann cells (Stassart et al., 2013), suggesting that soluble NRG1 plays a role also during the early phases of axonal degeneration and regrowth (Gambarotta et al., 2013). NRG1 receptors ErbB2, ErbB3 and ErbB4 are variably expressed by adult DRG sensory neurons and spinal cord MNs, and their levels increase although in a variable pattern after their axotomy, indicating that NRG1 proteins may act directly on the neurons (Pearson and Carroll, 2004). In our *in vitro* organotypic cultures, addition of exogenous NRG1 promoted the initial phase of neurite growth, likely acting on the neuronal processes. Accordingly, increased supply of exogenous NRG1 was shown to promote nerve regeneration *in vivo* (Chen et al., 1998; Nicolino et al., 2003; Joung et al., 2010). Our group has also recently reported that intramuscular administration of NRG1 Type 1 enhances the emergence of collateral sprouts of motor axons that reinnervate previously denervated muscle fibers in wild type and SOD1^G93A^ mice (Mancuso et al., 2016).

Another relevant aspect in the issue is whether the NRG1-ErbB pathway participates in the regulation of the neuroinflammatory response mediated by microglial cells, a common feature that occurs in human patients as well as in murine models of neurodegeneration. Activated ErbB2 receptors predominantly present on microglia and, to a lesser extent, on astrocytes were found overexpressed as a function of disease progression in the SOD1^G93A^ transgenic mouse, correlating with the pattern of microglial activation (Song et al., 2012). These findings led to suggest that NRG1 isoforms could contribute to disease pathogenesis through glial cell activation. Chronic excitotoxicity also induced a marked microglial response in the SCOC that was markedly reduced by administration of rhNRG1. Furthermore, we observed that microglial cells showed thinner and more ramified processes under rhNRG1 treatment compared to THA alone where microglia had an ameboid morphology and larger size. These results suggest that in this chronic excitotoxic model, rhNRG1 modulates the ErbB2 receptor expressed in microglia. Indeed, NRG1 signaling via the ErbB2 receptor was shown to be involved in microglial proliferation and chemotaxis after peripheral nerve injury (Calvo et al., 2010).

Recently, it has been reported that autophagy might influence inflammation and activation of microglia, as well as inflammation might promote or inhibit the process of autophagy (Su et al., 2016). Interestingly, IGF-I has been described to protect hippocampal neurons against early excitotoxicity via the NR2B/PI3K-AKT-mTOR pathway, suppressing the excess of autophagy (Wang et al., 2014). Considering these links between the different pathogenic mechanisms we hypothesize that rhNRG1 reduces the autophagy caused by excitotoxicity activating the ErbB receptors and the PI3K/AKT pathway in both MNs and microglial cells. This activation promotes a dual effect; the survival of the MNs and the reduction of the neuroinflammatory response mediated by the microglia. Therefore, the modulation of the expression of NRG1 at the central nervous system seems an interesting approach for promoting neuroprotection and maintenance of connectivity of the MNs.

## Author Contributions

GM-C carried out neuroprotection studies and wrote the manuscript. DS performed neurite growth studies. XN and MH-G made the experimental design, helped to perform the experiments, analyzed the data and wrote the manuscript.

## Conflict of Interest Statement

The authors declare that the research was conducted in the absence of any commercial or financial relationships that could be construed as a potential conflict of interest.

## Footnotes

^1^http://rsb.info.nih.gov/ij/
